# Tables for Doctor and Druggist

**Published:** 1891-10

**Authors:** 


### Book Reviews.

TABLES FOR DOCTOR AND DRUGGIST. Compiled by Eli H. Long, M. D., Professor of Materia Medica, Buffalo College of Pharmacy: Published by Geo. S. Davis, Detroit, Mich. Price $2.00.

This exceedingly valuable little book is just before us and its contents will be of inestimable value to the physician and druggist alike. It comprises the following divisions:

I. Table of solubilities.

II. Table of reactions and incompatibles.

III. Tables of doses and uses of medicines.

IV. Table of specific gravities.

V. Tables of poisons and antidotes.

Every physician will find this a suitable little book for his desk, and to the young physician that portion devoted to reactions and incompatibles will itself be worth the price of the book.

